# The Effect of *Ascophyllum nodosum* Extract on the Nutraceutical Antioxidant Potential of *Vigna radiata* Sprout under Salt Stress

**DOI:** 10.3390/plants10061216

**Published:** 2021-06-15

**Authors:** Sangeeta Kumari, Deepak Phogat, Krishnan D. Sehrawat, Ravish Choudhary, Vishnu D. Rajput, Jyoti Ahlawat, Rohini Karunakaran, Tatiana Minkina, Anita R. Sehrawat

**Affiliations:** 1 Department of Botany, Maharshi Dayanand University, Rohtak 124001, India; sangeetaghalawat@gmail.com (S.K.); j.10dec@gmail.com (J.A.); 2 Sarvodya College of Pharmacy, Imlota 127306, India; deepakphogat29@gmail.com; 3 Department of Genetics and Plant Breeding, CCS Haryana Agricultural University, Hisar 125004, India; krishanssehrawat@gmail.com; 4 Division of Seed Science and Technology, ICAR-Indian Agricultural Research Institute, New Delhi 110012, India; 5 Academy of Biology and Biotechnology, Southern Federal University, 344090 Rostov-on-Don, Russia; rvishnu@sfedu.ru (V.D.R.); tminkina@mail.ru (T.M.); 6 Unit of Biochemistry, Faculty of Medicine, AIMST University, Semeling, Bedong 08100, Kedah, Malaysia; rohinik23@gmail.com

**Keywords:** germination, sprouts, salinity, *Ascophyllum nodosum*, antioxidants

## Abstract

Mung bean (*Vigna radiata* L.) sprout is a popular fresh vegetable, tasty and high in antioxidants. To increase yield and quality after the occurrence of both abiotic and biotic stresses, the application of seaweed extracts is of great importance. Hence, this study was conducted to determine the effect of *Ascophyllum nodosum* extract (ANE) in the presence of salt on the antioxidant potential of *V. radiata* sprouts. Different concentrations of ANE viz. 0.00, 0.01, 0.05, 0.10, and 0.50% and NaCl 0, 25, 50, 75, and 100 mM alone and in combinations were tested for researching the antioxidant potential of *V. radiata* sprouts at 0, 24, and 36 h of sprouting. The DPPH free-radical-scavenging activity of sprouts of *V. radiata* was found to increase with time and peaked at 24 h of treatment. The *A. nodosum* extract (0.01%) could reverse the ill effect of the low level of salinity posed by up to 25 mM NaCl. The increasing salinity deteriorated the antioxidant activity using ABTS method of sprouts down to 20.45% of the control at 100 mM NaCl. The total phenolic content (TPC), total flavonoid content (TFC), and reducing power of *V. radiata* sprouts was found to increase till 36 h of sprouting. A slight increase in TPC, TFC and reducing power was observed when seeds were treated with low concentrations of ANE. The elevation in TPC, TFC and reducing power upon treatment with low concentrations of ANE was also noticed in sprouts in saline combinations. Alpha amylase inhibition activity was found to reach a (67.16% ± 0.9) maximum at 24 h of sprouting at a 0.01% concentration of ANE. Tyrosinase inhibition and alpha glucosidase inhibition was 88.0% ± 2.11 and 84.92% ± 1.2 at 36 h of sprouting, respectively, at 0.01% concentration of ANE. *A. nodosum* extract is natural, environmentally friendly, and safe, and could be used as one of the strategies to decline stress at a low level and enhance the antioxidant activities in *V. radiata* sprouts, thus increasing its potential to be developed as an antioxidant-based functional food.

## 1. Introduction

Beans are rich in nutrients (protein, carbohydrate, dietary fiber, and vitamins), micronutrients (potassium, magnesium, folate, iron, and zinc) as well as bioactive compounds such as polyphenols [1,2]. In addition to a protein sources, beans protein can be a source of biologically active peptides with antioxidant and anti-inflammatory properties [3]. Legume-based diets have long-term beneficial effects on human health, including a preventive effect against hypertension, cardiovascular disease, diabetes, and some types of cancer. Mung bean (*Vigna radiata* L.) is a popular leguminous crop accepted all over the world for its physiological functionalities, such as antitumor, antioxidant, and antidiabetic [4,5,6]. It is rich in vitamins, minerals, and proteins (25–28%) with high content of essential amino acids [7].

Food demand is expected to increase from 59% to 98% by 2050 [8]. The sustainable production of food requires new approaches to enhance the use efficiencies of all inputs, including nutrients. These approaches are vital to not only enhance agricultural productivity, but also to protect quality [9]. The monotonous consumption of cereals leads to a deterioration in overall nutritional status. Legumes are a major source of protein, but stress conditions affect the development and physiological, biochemical, morphological, and molecular integrity. Oxidative stress occurs when there is an imbalance between the production of reactive oxygen species (ROS) and antioxidant defense in any cellular compartment. Antioxidants are vitamins and minerals that occur naturally in foods and are also manufactured by our bodies [10]. Many naturally occurring antioxidants from plant sources have been identified as free radical scavengers or active oxygen scavengers [11,12]. Antioxidants are critical to human health. The property of antioxidants to quench the ROS marks their importance both for consumers and nutritionists.

Excessive ROS production in the human leads to oxidative damage towards fat, protein, and DNA [13,14]. Therefore, antioxidant-rich food is needed to keep the balance of ROS. Germination has been reported to enhance the nutritional value of *V. radiata* by reducing anti-nutritional and indigestible factors while increasing free amino acids, organic acids, phenolic compounds [15,16]. Germination increases antioxidant capacity in legumes [17]. *V. radiata* is a popular fresh vegetable consumed widely in the world [18] and its sprout has been reported to have antihypertensive, antidiabetic, and anticancer effects [19,20,21]. Therefore, the *V. radiata* sprout can be a valuable functional vegetable with potential in the prevention of chronic diseases. It was noted that sprouts have increased ascorbic acid and total phenolic content compared to *V. radiata* seeds; however, reports are inconsistent about the dynamic changes of their contents at different germination times [22,23]. Elicitation during germination is one of the factors which affect the increase of antioxidants in sprouts.

The nutraceutical properties in the *V. radiata* correlated to the presence of vitamins, flavonoids, and phenolics, as it is rich in polyphenolics, and the major phenolic constituents in *V. radiata* are phenolic acids, flavonoids, and tannins. [24]. The proteins, polypeptides, polysaccharides, and polyphenols from the seeds, sprouts, and hulls of *V. radiata* all show potential antioxidant activity [6]. The nutraceutical properties of the legumes are a result of the activity of free radical scavenger molecules [11,12]. *V. radiata* extracts possess significantly higher radical scavenging activities, greater reducing power, and higher levels of polyphenols than soybean extracts, suggesting that they are superior functional foods [6]. During the sprouting process, sprout extracts show higher amounts of total phenolics, total flavonoids, and DPPH radical-scavenging activity than seed extracts [25]. ABTS^+^ and DPPH radical cation assays were used for the evaluation of free-radical-scavenging properties of the 20 mung bean cultivars, and the antioxidant activity of 70% ethanol extracts of the mung beans showed strong DPPH and ABTS^+^ free-radical-scavenging capacity [24]. Legumes are rich source of tyrosinase inhibitors [26]. A comparison of the anti-tyrosinase activity of various legumes by Yao et al. [27] revealed *V. radiata* as an important source of anti-tyrosinases. The *V. radiata* sprouts exhibited considerable anti-tyrosinase activity, such that the sprouts were recommended to be used in the cosmetic industry [28]. Legumes are also known to possess anti-diabetic properties, and this property of the legumes is attributed to their ability to inhibit the activity of α-glucosidase activity and α-Amylase enzymes that are involved in the hydrolysis of carbohydrates [5]. Aqueous and ethanolic extracts of the *V. radiata* showed a consequential inhibitory effect on the starch-hydrolyzing enzymes, such as gastrointestinal α-amylase (pancreatic) and α-glycosidase (intestinal) [29]. *V. mungo* (black mung bean), Jiheilv 27-3 exhibited significant anti-diabetic activity than other varieties of the pulse. An inhibitor of the enzymes competes for its active site to control its activity, thereby slowing down the catabolism of carbohydrates and preventing increasing blood glucose levels [30]. 

Crop plants are generally exposed to a series of abiotic and biotic stresses. Abiotic stresses disturb plant osmosis, imbalance nutrition channels, and create ionic toxicity [31,32]. The alteration in these main mechanisms leads to metabolic and physiological changes. It poses a negative impact on seed germination and seedling growth, as evident from retarded shoot and root length, fresh and dry weight, chlorophyll content, and synthesis [33,34,35]. Salinity is one of the predominant abiotic stresses that affect agricultural production. The adverse effects of salinity have damaged at least 20% of crop cultivations worldwide [36]. Salinity stress causes severe yield loss (>70% even at 50 mM sodium chloride) and affects the quality of *V. radiata* [37,38]. Salinity affects almost all growth stages of plant development [39,40,41,42,43]. Decreases in germination, shoot and root lengths, and fresh mass in *V. radiata* under NaCl were studied by Promila and Kumar [44]. The accumulation of sodium and chloride ions leads to poor activation of the hydrolytic enzymes and results in reduced seed germination in the saline environment [45,46]. To overcome the salt stress, plants need to improve ion exclusion, osmotic tolerance, redox homeostasis, and efficient photosynthesis. 

Seaweed extract has been reported to alleviate a variety of abiotic stresses, including drought, salinity, and temperature [47], and are inexpensive and easy to prepare and use [48]. *Ascophyllum nodosum*, being a bio-fertilizer, may support the growth of *V. radiata*, similar to other seaweeds [49]. *A. nodosum* may provide a healing touch to the salinity-stress-induced damage on the plants. It is one of the most commonly used seaweeds. The effect of seaweed is mediated via an intricate network of signals that perceive stress and set in motion molecular, biochemical, and physiological processes that may be unique to each stress [50]. Increasing plant resistance to stressors through the use of biostimulants is likely due to changes in enzymatic activity and increased synthesis of antioxidative compounds [51]. Polysaccharides and polyphenols are present in the extract due to their allelochemical potential and capability to increase plant resistance to stress conditions [52]. Fan et al. [53] observed an increase in total protein content, antioxidative capacity, and phenolic and flavonoid contents in spinach treated with *A. nodosum* extract. It also increases the endogenous concentrations of stress-related molecules, such as cytokinin’s, proline, and antioxidants in treated plants [53]. Hence, this study aimed to determine the effect of *A. nodosum* extract and NaCl alone and in combination on some biological antioxidant potential of *V. radiata* sprouts.

## 2. Results

### 2.1. Effect on DPPH Free-Radical-Scavenging Activity

**Figure 1:**
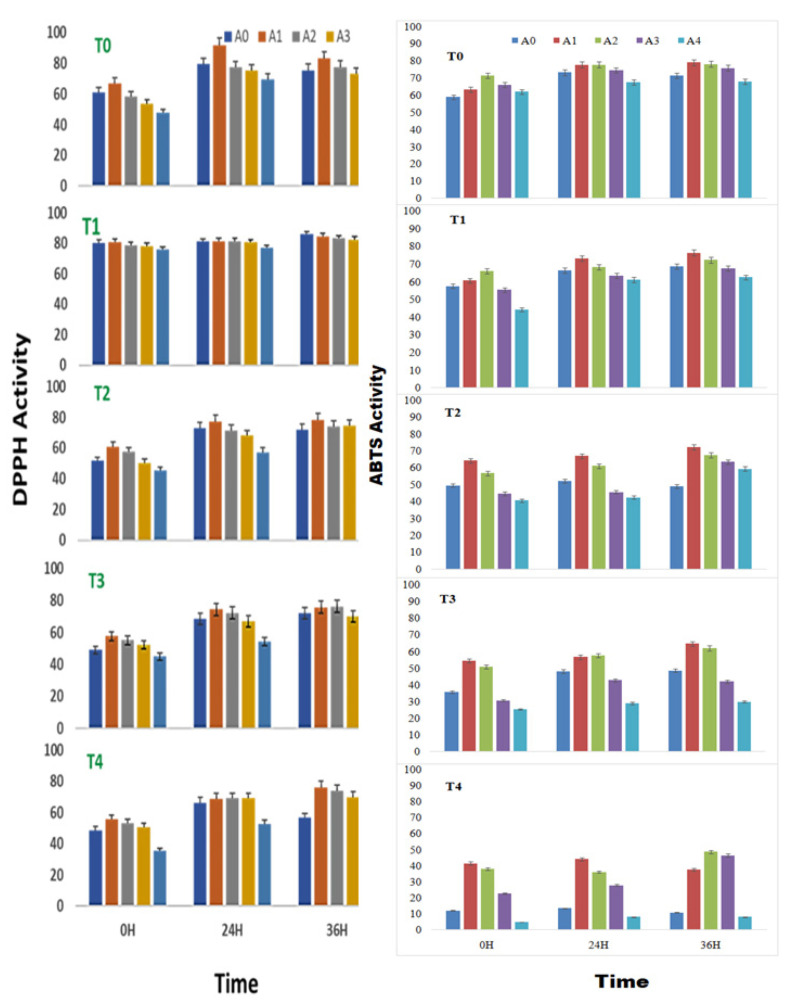
DPPH and ABTS radical-scavenging activity (% inhibition) of sprouts of *Vigna radiata* under salt stress in the presence of *A. nodosum* extract (ANE). (A0–A4 are ANE concentrations of 0.00, 0.01, 0.05, 0.10, and 0.50%, and T0–T4 are NaCl concentrations of 0, 25, 50, 75 and 100 mM, respectively). Bar indicates mean ± SD (*n* = 3).

The DPPH radical-scavenging activity of sprouts of *V. radiata* was found to increase with time and peaked at 24 h of treatment in control and at 0.01% of ANE. The treatments, T2 T3 and T4 with ANE, were superior at 36 h of sprouting. In a stress-free environment, 0.01% ANE treatment positively influenced the DPPH radical-scavenging activity (91.8%) at 24 h of sprouting. At 0 h of treatment, the DPPH radical-scavenging activity was increased from 61.1 ± 0.3% to 67.1 ± 0.3% in the radical scavenging of sprouts when treated with 0.01% ANE. Similarly, at 24 h of treatment, the DPPH radical-scavenging activity was increased from 79. 6% ± 0.4% to 91.8 ± 0.3% in the sprouts with 0.01% ANE treatment; and at 36 h, the DPPH radical-scavenging activity was increased from 75.6 ± 0.4% to 83.2 ± 0.4% with 0.01% ANE. However, treatment with more than 0.05% ANE had a negative effect on the DPPH radical-scavenging activity (Figure 1).

A high concentration of ANE (0.50%) alone or in combination with NaCl showed the least DPPH radical scavenging activities. Salinity had a visible toll on the DPPH radical-scavenging activity. The DPPH radical scavenging was reduced from 61.1 ± 0.3% in control to 48.6 ± 0.2% in the sprouts stressed under the influence of salinity induced by 100 mM NaCl. Likewise, after 24 h of treatment, the DPPH radical-scavenging activity was reduced from 79.7 ± 0.4% in control to 66.4 ± 0.3% in the sprouts under 100 mM of NaCl-induced salinity; while DPPH radical-scavenging activity was reduced from 75.6 ± 0.4% in control to 56.7 ± 0.3% in the sprouts stressed under 100 mM of NaCl. 0.01%. ANE could reverse the ill effect of the low level of salinity posed by up to 50 mM NaCl (Figure 1).

During high salt stress, the 0.01% ANE did reverse the effect of the salinity but could not restore the native levels. The application of 0.01% ANE to seed sprouts stressed with 50 mM NaCl increased the DPPH radical-scavenging activity from 51.6 ± 0.2% to 60.7 ± 0.3% at 0 h of treatment, while the 0.01% ANE application to sprouts at 50 mM NaCl increased the DPPH radical-scavenging activity from 72.9 ± 0.4% to 77.4 ± 0.4% at 24 h of treatment and, 0.01% ANE application to sprouts at 50 mM NaCl increased the DPPH radical-scavenging activity from 72.1 ± 0.3% to 78.3 ± 0.4% at 36 h of treatment.

Two-way ANOVA analysis revealed that the differences in the DPPH radical-scavenging activity of sprouts of *V. radiata* due to the changes in the ANE concentrations (with F = 9.12, *p* < 0.0005 for 0 h; F = 8.19, *p* < 0.001 for 24 h; and F = 4.22, *p* < 0.05 for 36 h), and NaCl concentrations (with F = 28.41, *p* < 0.00001 for 0 h; F = 17.09, *p* < 0.00001 for 24 h; and F = 9.24, *p* < 0.0005 for 36 h) were significant (Table 1).

### 2.2. Effect on ABTS Free-Radical-Scavenging Activity

*A. nodosum* extract treatment was found to have a positive effect on the ABTS radical-scavenging activity of *V. radiata*, even in the absence of any saline stress. Treatment of *A. nodosum* to the sprouting stages of *V. radiata* seeds amended the ABTS radical-cation-scavenging activity till the concentration of 0.05% was achieved. The effect was more evident at 0 h of treatment. The ABTS radical-cation-scavenging activity was increased to 71.4 ± 1.6% inhibition with 0.05% ANE treatment against 58.9 ± 1.3% inhibition in the corresponding control at 0 h of treatment. Similarly, after 24 h, the ABTS radical-cation-scavenging activity showed 73.2 ± 1.7% inhibitions. The application of 0.01 and 0.05% ANE increased the ABTS radical-cation-scavenging inhibition to 77.7± 1.8%, and after 36 h, the application of 0.01% ANE increased the ABTS radical-cation-scavenging inhibition to 79.0 ± 1.8% against 71.4 ± 1.61% in control. 

The ANE (0.01% and 0.05%) showed maximum ABTS radical-cation-scavenging activities (79.0% and 78.1% followed by 71.7% and 74.6% at 24 and 36 h, respectively of seed soaking (Figure 1). An increase in the salinity deteriorated ABTS radical-cation-scavenging activity at all tested periods (0 h, 24 h and 36 h) of sprouting. A 58.9 ± 1.3% inhibition activity in control at 0 h to 12.1 ± 0.3 in T4 at 0 h were observed. Similarly, the ABTS radical-cation-scavenging activity at 24 and 36 h remained only 73.2% and 71.4%, respectively, at control and decreased (13.4% and 12.7% at the same period of sprouting). However, ANE, especially at concentrations of 0.01% and 0.05%, had a healing effect to the increasing saline stress. With the application of 0.01% and 0.05% ANE, the ABTS radical-cation-scavenging activity remained at 65.5% and 53.0%, 56.9% and 46.5%, and 47.5% and 62.3% after 0, 24, and 36 h of treatment in the sprouting stages of *V. radiata*. A two-way analysis of variance was employed to estimate the significance of the observed difference due to ANE application to salinity-stressed sprouts and the changes induced due to salinity stress and ANE applications were found significant with a *p*-value of less than 0.0001 (Table 2).

### 2.3. Effect on Total Phenol Content (TPC)

**Figure 2:**
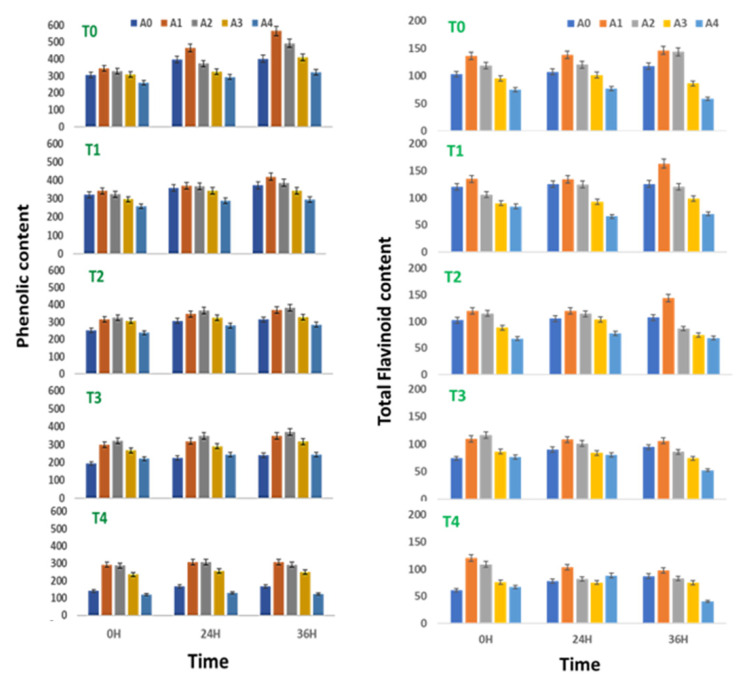
Phenolic (µg GAE) and total flavonoid contents (µg QE) in sprouts of *V. radiata* under salt stress in the presence of *A. nodosum* Extract (ANE). (A0–A4 are ANE concentrations 0.00, 0.01, 0.05, 0.10, and 0.50% and T0–T4 are NaCl concentrations 0, 25, 50, 75 and 100 mM, respectively). Bar indicates mean ± SD (*n* = 3).

The phenol content of sprouts of *V. radiata* was found to increase with time till 36 h of treatment. Treatments of 0.01% and 0.05% ANE showed maximum phenol accumulation at 36 h of sprouting followed by 24 h. A similar trend of phenol accumulation was observed when ANE was used in various combinations of NaCl. In a stress-free environment, ANE treatment at concentrations up to 0.1% increased the phenol content of the sprouting stages as compared with the sprouts devoid of ANE (Figure 2). The phenol content of the *V. radiata* sprouts was 305.9 ± 14.9 µg GAE fresh weight at 0 h, which was increased to 344.9 ± 16.75 µg GAE fresh weight with 0.01% ANE treatment. 

The increasing ANE concentration decreased the phenolic content that reached the basal level when the ANE concentration reached 0.01% and below basal level with the treatment of 0.05% ANE. The phenol content at 24 h of the *V. radiata* sprouts was 396.4 ± 19.3 µg GAE fresh weight which was increased to 465.8 ± 22.7 µg GAE fresh weight with the 0.01% ANE treatment and the higher concentration of ANE reduced the phenol content below the basal level. Similarly, the phenol content of the *V. radiata* sprouts after 36 h of treatment was 402.2 ± 19.6 µg GAE fresh weight, which was increased to 565.9 ± 27.5 µg GAE fresh weight with the 0.01% ANE treatment. The increasing ANE concentration decreased the phenolic content that reached the basal level when the ANE concentration reached 0.01% and below basal level with the treatment of 0.05% ANE (Figure 2).

At 0 h of treatment, the salinity stress created by 100 mM NaCl reduced the total phenol content from 305.9 ± 14.9 µg GAE fresh weight of control to 140.8 ± 6.9 µg GAE fresh weight in *V. radiata* sprouts. Similarly, at 24 h, the total phenol content decreased from 396.4 ± 19.3 µg GAE fresh weight of control to 167.8 ± 8.2 µg GAE fresh weight at 100 mM NaCl, and after 36 h, 402.2 ± 19.6 µg GAE fresh weight of control to 167.8 ± 8.2 µg GAE fresh weight at 100 mM NaCl. 

ANE treatment was found to exert a healing effect on the salinity-stress-induced changes in the phenol content. At 0 h, the 100 mM NaCl-induced phenolic level of 140.8 ± 6.9 µg GAE fresh weight was increased to 292.6 ± 14.3 µg GAE fresh weight when treated with 0.01% ANE. Similarly, at 24 h, the 100 mM NaCl-induced phenolic level of 167.3 ± 8.1 µg GAE fresh weight was increased to 308.4 ± 14.9 µg GAE fresh weight when treated with 0.01% ANE, while at 36 h, the 100 mM NaCl-induced phenolic level of 167.8 ± 8. µg GAE fresh weight was increased to 306.9 ± 14.9 µg GAE fresh weight when treated with 0.01% ANE (Figure 2). 

Two-way ANOVA analysis revealed that the differences in the phenol content of sprouts of *V. radiata* due to the changes in the ANE (with F = 12.635, *p* < 0.0005 for 0 h; F = 8.811, *p* < 0.001 for 24 h; and F = 10.879, *p* < 0.00001 for 36 h) and NaCl concentrations (with F = 9.923, *p* < 0.00001 for 0 h; F = 11.591, *p* < 0.0005 for 24 h; and F = 19.1399, *p* < 0.00001 for 36 h) were significant (Table 3).

### 2.4. Effect on Total Flavonoid Content (TFC)

The flavonoid content of the sprouting stages of *V. radiata* seeds growing in a stress-free environment was found to increase with time till 36 h of treatment. The flavonoid content of the sprouts was found to elevate upon treatment with 0.01 and 0.05% of the ANE in all the three time periods studied, i.e., 0, 24, and 36 h of treatment. At 0 h of treatment, the flavonoid content was 103.3 ± 5.1 µg QE fresh weight, which was increased to 136.5 ± 6.7 µg QE fresh weight when treated with 0.01% ANE. The values decreased thereafter, with an increase in the ANE concentration to reach the basal level at 0.1% ANE. 

Similarly, at 24 h of treatment, the flavonoid content was 107.3 ± 5.3 µg QE fresh weight which was increased to 138.2 ± 6.8µg QE fresh weight when treated with 0.01% ANE. The values decreased thereafter, with an increase in the ANE concentration to reach the basal level at 0.1% ANE. At 36 h of treatment, the flavonoid content was 117.7 ± 5.6 µg QE fresh weight, which was increased to 146.3 ± 5.8 µg QE fresh weight when treated with 0.01% ANE. The values decreased thereafter, with an increase in the ANE concentration.

The salinity stress takes a toll on the flavonoid content, except at 25 mM NaCl (Figure 2). The flavonoid level was reduced to 61.4 ± 3.0 µg QE fresh weight at 100 mM NaCl against 103.3 ± 5.1 µg QE fresh weight in the control sprouts at 0 h of treatment. Similarly, at 24 h of treatment, the flavonoid level was reduced to 78.3 ± 3.8 µg QE fresh weight at 100 mM NaCl against 107.7 ± 5.3 µg QE fresh weight in the control sprouts. Meanwhile, at 36 h of treatment, the flavonoid level was reduced to 87.6 ± 4.3 µg QE fresh weight at 100 mM NaCl against 117.7 ± 5.8 µg QE fresh weight in the control sprouts. 

The ANE treatment was found to reverse the salinity-mediated decrease of the flavonoid content (Figure 2). At 0 h of treatment, the 0.01% ANE treatment brought the flavonoid level at 100 mM NaCl to 120.4 ± 5.9 µg QE fresh weight. Further, the 0.05% ANE also brought the level to the basal level. Similarly, at 24 h of treatment, the 0.01% ANE treatment brought the flavonoid level at 100 mM NaCl to 103.9 ± 5.2 µg QE fresh weight. Meanwhile, at 36 h of treatment, the 0.01% ANE treatment brought the flavonoid level at 100 mM NaCl to 98.3 ± 4.5 µg QE fresh weight.

Two-way ANOVA analysis revealed that the differences in the flavonoid content of sprouts of *V. radiata* due to the changes in the ANE at 0 (F = 3.255, *p* < 0.05), 24 (F = 10.28, *p* < 0.0005) and 36 h (F = 25.35, *p* < 0.00001) and NaCl concentrations at all time points, i.e., 0 (F = 17.612, *p* < 0.05), 24 (F = 4.115, *p* < 0.05), and 36 h (F = 9.427, *p* < 0.0005) of treatment were significant (Table 4).

### 2.5. Effect on Reducing Power 

**Figure 3:**
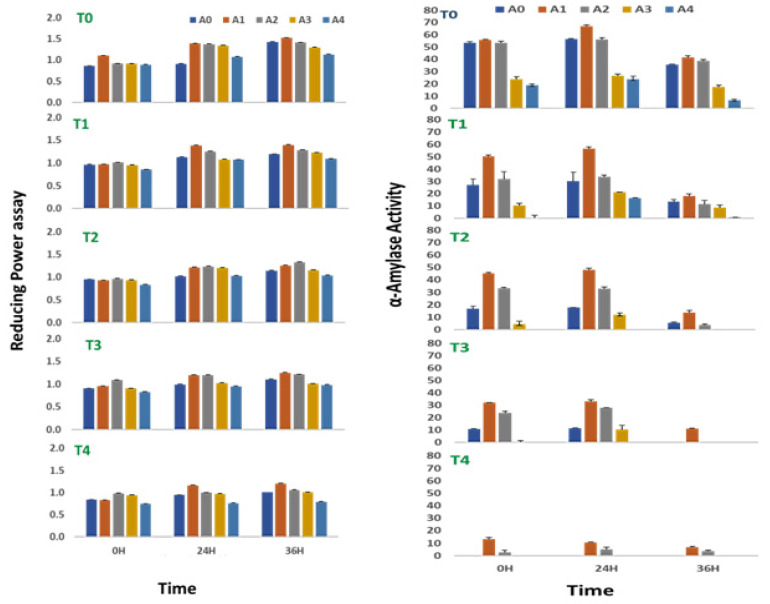
Reducing power assay and alpha amylase activity of sprouts of *V. radiata* under salt stress in the presence of *A. nodosum* Extract (ANE). (A0–A4 are ANE concentrations 0.00, 0.01, 0.05, 0.10, and 0.50% and T0–T4 are NaCl concentrations 0, 25, 50, 75, and 100 mM, respectively). Bar indicates mean ± SD (*n* = 3).

The reducing power of sprouts of *V. radiata* was found to increase with time till 36 h of treatment, irrespective of the treatment provided to the sprouts. The salinity stress below 100 mM NaCl was found to slightly elevate the reducing power of the sprouts. The reducing power of sprouts of the *V. radiata* was 0.86 ± 0.01 at 0 h of treatment, which was increased to 0.96 ± 0.01 at the salinity induced by 25 mM NaCl. The reducing power decreased with the further increase in the soil salinity and reached the basal level at 100 mM NaCl (Figure 3). Similarly, at 24 h, the reducing power of sprouts of the *V. radiata* was 0.91 ± 0.01, which was increased to 1.1 ± 0.01 at the salinity induced by 25 mM NaCl. The reducing power decreased with the further increase in salinity and reached the basal level at 100 mM NaCl. Meanwhile, the reducing power of sprouts of the *V. radiata* was 1.4 ± 0.01 at the 36 h of the treatment, which subsequently decreased with an increase in the soil salinity and reached 1.0 ± 0.01 at the salinity induced by 100 mM NaCl.

*V. radiata* sprouts in a stress-free environment were observed to have a slight increase in their reducing power when treated with low concentrations of ANE. The observation was more evident during the early hours of treatment. In all the experiments, the reducing power of the sprouts reached their peak values when treated with 0.01% of ANE, irrespective of the time periods studied or the concentration of the NaCl supplied to it (Figure 3). The reducing power of the *V. radiata* sprouts was found to increase from 0.83 ± 0.0 in the control of 100 mM NaCl to 0.98 ± 0.01 when treated with 0.01% ANE at 0 h of treatment, whereas the reducing power at 24 h was increased from 0.9 ± 0.01 in the control of 100 mM NaCl to 1.2 ± 0.01, and the reducing power at 36 h was increased from 1.0 ± 0.01 to 1.2 ± 0.01 in the 0.01% ANE treated sprouts.

Two-way ANOVA analysis revealed that the differences in the reducing power of sprouts of *V. radiata* were due to the changes in the ANE at all time periods, i.e., 0 (F = 5.322, *p* < 0.01), 24 (F = 7.33, *p* < 0.005), and 36 h (F = 34.100, *p* < 0.00001) after treatment, and NaCl concentrations at 24 (F = 11.77, *p* < 0.005) and 36 h (F = 29.58, *p* < 0.00001) of treatment were significant (Table 5).

### 2.6. Effect on α-Amylase Activity

The α-amylase activity of sprout of *V. radiata* seeds was found to reach its peak after 24 h of treatment. The *A. nodosum* treatment amended the α-amylase activity in the absence of saline stress in the sprouting stages. Providing low concentration (0.01%) of *A. nodosum* extract to the sprouting stages had a positive effect on the α-amylase activity. At 0 h, the alpha-amylase activity was 55.9 ± 1.2% with the supplementation of the 0.01% ANE against 55.4 ± 1.2% in control. Similarly, at 24 h, the alpha-amylase activity was 67.2 ± 0.9% with the supplementation of the 0.01% ANE against 56.7 ± 0.5% in control, and at 36 h, the alpha-amylase activity was 41.6 ± 0.7% with the supplementation of the 0.01% ANE against 35.6 ± 0.6% in control. 

An increase in salinity to the concentration of 100 mM NaCl completely abolished α-amylase activity at 0, 24, and 36 h of treatment (Figure 3). The ANE treatment, however, prevented the loss of the activity, and ~24% of the activity was retained at 0 h with the treatment of 0.01% of ANE. The activity was ~16% at 24 and 36 h of treatment with 0.01% ANE in sprouts growing in 100 mM NaCl saline stress. Two-way ANOVA analysis revealed the significance in the differences among the values obtained due to variations in enzyme activity at 0 (F = 34.99, *p* < 0.0001), 24 (F = 26.8, *p* < 0.0001), and 36 (F = 15.27, *p* < 0.0001) hours of the treatment for salinity and 0 (F = 39.32, *p* < 0.0001), 24 (F = 22.96, *p* < 0.0001), and 36 (F = 17.12, *p* < 0.0001) hours of the treatment for ANE (Table 6).

### 2.7. Effect on the α-Glucosidase Activity

The α-glucosidase activity of sprouting stages of *V. radiata* seeds increased with time throughout the experiment of 36 h. Treatment with a low concentration of *A. nodosum* extracts positively affected the α-glucosidase activity of sprouts in the absence of saline stress. An increase in the soil salinity to 100 mM NaCl abolished α-glucosidase activity to nearly half at 0, 24, and 36 h of treatment. At 0 h, the α-glucosidase activity was reduced to 23.5 ± 0.3% at 100 mM NaCl against 48.2 ± 0.7% in control. Similarly, at 24 h, the α-glucosidase activity was 28.6 ± 0.4% with the supplementation of 100 mM NaCl against 71.1 ± 0.9% in control, and at 36 h, the α-glucosidase activity was reduced to 37.8 ± 0.5% at 100 mM NaCl against 75.3 ± 1.04% in control. 

**Figure 4:**
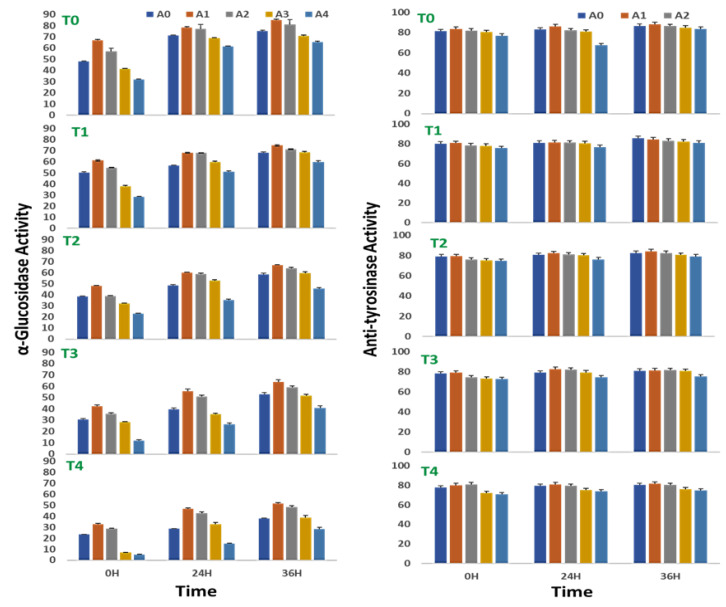
Alpha-glucosidase and tyrosinase inhibition of sprouts of *V. radiata* under salt stress in the presence of *A. nodosum* extract (ANE). (A0–A4 are ANE concentrations 0.00, 0.01, 0.05, 0.10, and 0.50% and T0–T4 are NaCl concentrations 0, 25, 50, 75 and 100 Mm, respectively). Bar indicates mean ± SD (*n* = 3).

The ANE treatment, however, minimized the loss of the activity to some extent at 24 and 36 h of treatment (Figure 4). At 0 h of treatment, 0.01% ANE improved the percent inhibition by α-glucosidase activity by 10–12 units over the corresponding control. Similarly, at 24 h, the α-glucosidase activity treatment with 0.01% ANE increased the percent inhibition to nearly 12–18 units over that of the control, and at 36 h of treatment, 0.01% improved the α-glucosidase activity to 6-14 units over that of the control. At 100 mM salinity, the 0.01% ANE treatment recovered the salinity-mediated impairment of α-glucosidase activity from 23.5 ± 0.3% to 32.8 ± 0.5% at 0 h, 28.6 ± 0.4% to 46.7 ± 0.6% at 24 h, and 37.8± 0.5% to 51.5 ± 0.7% at 36 h.

Two-way ANOVA revealed significances in the differences of the α-glucosidase activity of sprouts of *V. radiata* due to the changes in the *A. nodosum* and NaCl concentrations in the microenvironment of sprouts. The ANOVA yielded F = 75.33, 115.42, and 229.87 for 0, 24, and 36 h, respectively, and *p* < 0.0001 for salinity-induced differences, and F = 70.24, 46.75, and 77.05 for 0, 24 and 36 h, respectively, and *p* < 0.0001 for ANE-mediated differences (Table 7).

### 2.8. Effect on the Anti-Tyrosinase Activity 

The anti-tyrosinase activity of sprouts of *V. radiata* was found to marginally increase with time from 0 to 36 h. In a stress-free environment, a low concentration of ANE treatment did not affect the anti-tyrosinase activity of sprouts, though in the treatment with 0.5% ANE, it had a negative effect on the anti-tyrosinase activity (Figure 4). At 0 h of treatment, the anti-tyrosinase activity was reduced from 81.3 ± 1.9% in control to 76.9 ± 1.8 in 0.1% ANE-treated, stress-free sprouts. Similarly, at 24 h of treatment, the anti-tyrosinase activity was reduced from 82.9 ± 1.2% in control to 86.6 ± 2.1 in 0.1% ANE-treated, stress-free sprouts; and after 36 h of treatment the anti-tyrosinase activity was reduced from 86.6 ± 2.1% in control to 83.4 ± 2.0 in 0.1% ANE-treated, stress-free sprouts. 

Salinity negatively regulated the anti-tyrosinase activity of sprouts of *V. radiata* seeds. The anti-tyrosinase activity decreased to 77.7 ± 1.9% when grown in salinity due to supplementation of 100 mM NaCl against 81.3 ± 1.9% in the control sprouts at 0 h of treatment. Similarly, the anti-tyrosinase activity decreased to 79.4 ± 1.9% when grown -n salinity due to supplementation of 100 mM NaCl in the soil against 82.9 ± 1.9% in the control sprouts at 24 h of treatment, and the anti-tyrosinase activity was decreased from 86.6 ± 2.1% in the control sprouts to 80.4 ± 1.9% when grown in salinity due to supplementation of 100 mM NaCl in the soil at 36 h of treatment. Low concentrations (0.01 and 0.05%) of ANE treatment showed recovery, especially at the higher concentration (100 mM) of salt after 0 h of treatment. 

Two-way ANOVA analysis revealed that the changes in the tyrosinase activity were found to be highly significant at all three time periods studied (ANOVA; F = 9.07 *p* < 0.001 for 0 h, F = 10.86; *p* < 0.0005 for 24 h, and F = 19.59; *p* < 0.00001 for 36 h) (Table 8). However, the effect of ANE concentrations in the microenvironment on the anti-tyrosinase activity of sprouts of *V. radiata* was significant at 0 h (ANOVA, F = 13.17; *p* < 0.00001) and 36 h (ANOVA, F = 37.48; *p* < 0.00001) of treatment, while non-significant at 24 h (ANOVA, F = 1.03; *p* = NS) (Table 8).

## 3. Discussion

The nutraceutical potential of legumes is linked with a high content of bioactive constituents, such as phenolics, vitamins, and bioactive peptides [54]. Germination of *V. radiata* caused an increase in both DPPH radical-scavenging activity and reducing power of sprouts. The increase in antioxidative activity may be due to the increase in the amount of polyphenolics content, antioxidative amino acids, and some peptides. Phenolic functional groups serve as free radical sequestrants [55]. Kim et al. [25] found the highest DPPH radical-scavenging activity in *V. radiata* sprouts rather than seeds. The antioxidant activity of *V. radiata* sprouts was six times higher than seeds [22]. Zhaohui et al. [56] also reported increased antioxidant activity in germinated *V. radiata*, *Glycine max*, and *Phaseolus vulgaris* sprouts. Gan et al. [57] found increased ascorbic acid, total phenolic content, and antioxidant capacity in *V. radiata* upon germination. Marathe et al. [58] showed potential antioxidant activity of *V. aconitifolia*. Application of Kelpak Seaweed Liquid, Terra Sorb Complex caused an increase in radical-scavenging activity via ABTS assay in all tested combinations as compared to the control [59]. Huang et al. [23] found that the antioxidant capacity of the dry *V. radiata* sprouts gradually increased during a 5-day and 9-day germination time.

Plant phenolics are very desirable compounds in the human diet because of their antioxidant properties and different preventative roles against diseases associated with oxidative stress, such as cancer and cardiovascular and neurodegenerative diseases [60]. Therefore, changes in phenolic concentrations may influence the antioxidant activities of plant foods. Seaweed extract can also influence plant metabolism, enhancing the content of some bioactive compounds. Pise and Sabale [61] studied the effect of three seaweed extracts (*Ulva fasciata, Sargassum ilicifolium*, and *Gracilaria corticata*) on the yield and quality of *T. foenum-graecum* and found significantly higher phenolic contents in plant sprayed with the tested extracts. Kocira et al. [62] observed that the total phenolics content was increased in soybean seeds after 1.0% Fylloton biostimulant treatment. Fernandez-Orozco et al. [63] found that during germination of *V. radiata*, the total phenolic content increased from 1.1 to 1.4 and 3.5 mg/g after germination for 2 and 7 days, respectively. Ertani et al. [64] reported the increased contents of phenols and flavonoids in corn plants grown under stress conditions. 

The accumulation of phytochemicals compounds and antioxidant activity could be increased by the application of brown seaweed extract, thus enhancing the nutritional values of treated plants [65,66]. Higher contents of phenolic compounds and antioxidants in brown seaweed extract in plants might affect the metabolic processes of treated plants, consequently leading to an accumulation of phytochemical compounds and increasing antioxidant activity; accordingly, the nutritional values of the treated plants were enhanced [66,67,68]. Rhocha-Guzman et al. [69] reported that there was a high correlation between total phenolic contents and DPPH radical-scavenging activity.

The highest content of flavonoids in seeds of *Glycine max* was found after a single application of a biostimulant [62]. According to Fan et al. [70], commercial extracts of *A. nodosum* at 1.0 g/L significantly enhanced the total phenolic and flavonoid contents and antioxidant activity in spinach leaves. Plants treated with extracts from *Ecklonia maxima* had an increase in flavonoid content of bean seeds compared to the control treatment [59]. Hand et al. [71] observed lower total flavonoid content in pepper at higher NaCl concentrations. The effect of NaCl concentration, treatment time, and their interaction showed significant changes in total flavonoid content. Treated plants of *Spiraea nipponica* with *Pittosporum eugenioides* showed higher phenolic and flavonoid contents under mild drought conditions [72].

The antioxidant capacity of aura seeds was significantly improved by the application of *Ecklonia maxima* Kelpak seaweed and the reducing power and antiradical ability were increased. The results for reducing potential were about 50% higher for all variants of application in respect to control [62]. The reducing power in *G. max* was determined by Kocira et al. [62] by foliar application of Fylloton; almost all applied biostimulant combinations increased the value of the reducing power. The highest reducing power was found after double-spraying with a 0.7% biostimulant in the Mavka cultivar and a 27.0% increase in the Annushka cultivar as compared to the control. Orak et al. [73] observed that the reducing antioxidant power of extracts and seeds of *V. radiata* were higher in acetone extract than methanol extract. The reducing power of the *V. radiata* extract was significantly higher than that of the soybean extracts [74,75].

The α-amylase catalyzes the hydrolysis of glycosidic linkages in the starch and releases the hydrolyzed products, which constitute the first step in the enzymatic degradation of this polymer. α-Amylase inhibitors can bind with the reactive sites of α-amylase enzyme and alter its catalytic activity, and thus reduces the blood sugar level. Hence, at present, there is an increasing interest among food scientists to search for an alternative natural source of an α-amylase inhibitor with potential antioxidant activity [76,77,78]. Vadivel et al. [79] observed that the methanolic extract of raw seed of *S. sesban* showed 81.43% of α-amylase inhibition. This value is comparable with (87%) that of Mucuna *pruriens* seeds [80] and also higher (65%) than *V. radiata* [81]. The methanolic extract of raw seed materials of *Acacia nilotica* showed 72.91% of α-amylase inhibition [30]. Paradis et al. [82] found the two brown seaweeds (*A. nodosum* and *Fucus vesiculosus*) potentially inhibit α-amylase and α-glucosidase without any adverse effect. Sprouting of soybeans showed a similar ability to increase extract anti-amylase activity, which was highest after 4–6 d of germination time [83]. *Hizikia fusiforme*, *Capsosiphon fulvescens*, *Undaria pinnatifida* sporophyll, and *U. pinnatifida* blade extracts showed weak, 26.7, 30.1, 22.7, and 33.6%, α-amylase inhibitory activities, respectively, at a concentration of 10 mg/mL. On the contrary, apart from *C. fulvescens*, good performance of all seaweed extracts was observed for the suppression of α- glucosidase, suggesting the seaweed extracts specifically targets α-glucosidase [16]. Ranilla et al. [84] and Zhang et al. [85] revealed that extracts rich in phenolic compounds had weak or no inhibition effects against α-amylase and strong inhibition effects against α-glucosidase.

The α-glucosidase is one of the glucosidases located at the epithelium of the small intestine. The α-glucosidase has been recognized as a therapeutic target for modulation of postprandial hyperglycemia, which is the earliest metabolic abnormality to occur in type 2 diabetes mellitus. Inhibition of intestinal α-glucosidases delays the digestion and absorption of carbohydrates, thereby suppressing postprandial hyperglycemia. α-glucosidase inhibitors are oral anti-diabetic drugs used for diabetes mellitus type 2 that work by preventing the digestion of carbohydrates [86]. The increased free radical production and reduced antioxidant defense may partially mediate the initiation and progression of diabetes-associated complications. Thus, antioxidant activity and anti-diabetic properties of legumes are related. Methanolic extract of raw seed materials of *Sesbania sesban* showed 67.05% of α-glucosidase enzyme inhibition [79]. Randhir et al. [87] studied *Triticum* sp., *Fagopyrum esculentum*, *Zea mays*, and *Avena sativa* and observed an (18–31%) α-glucosidase enzyme inhibition. Kim et al. [88] and Shobana et al. [89] studied *Setaria italica*, *Panicum miliaceum*, *Sorghum bicolor*, and *Eleusine coracana*, and found 82.5%, 77%, 95%, and 78% α-glucosidase enzyme inhibition activity, respectively. *Psoralea corylifolia* seeds have 77.5% α-glucosidase enzyme inhibition activity [90]. Burguieres et al. [91] reported that in peas, upon germination, increased phenolic-enriched content was more effective in controlling alpha-glucosidase in relation to hyperglycemia. Sreerama et al. [92] studied the phenolic extracts of four types of beans and found the biggest influence in terms of alpha glucosidase inhibition in *V. angularis* (black adzuki bean) and *V. radiata* which showed the least activity. 

Tyrosinase is the enzyme involved in melanogenesis and catalyzes the oxidation process of tyrosine to dihydroxy-phenylalanine (DOPA) and from DOPA-to-DOPA quinone. Tyrosinase is known to be a metalloenzyme, containing copper at an active site, catalyzing these reactions through a change in the oxidative site of copper atoms [93]. Tyrosinase is a copper-containing enzyme that catalyzes the rate of melanin synthesis and other pigments from tyrosine by oxidation. Although the melanin pigmentation is important in human skin and acts as a major defense mechanism against the ultraviolet light of the sun, unbalanced production, such as in the form of freckles, age-spots, and other forms of melanin hyperpigmentation, can lead to serious problems [94]. 

Natural products containing melanin synthesis inhibitory activity are of interest, with their potential cosmetic applications, for example, in skin-whitening or anti-browning preparations. Four plant extracts in three different organic solvents and deionized water exhibited relatively high tyrosinase inhibition levels as follows: 975.7% in *Citrus sinensis* (ethyl acetate), 690.4% in *Vitis vinifera* (deionized water), 509.2.% in *Fragaria ananassa* (ethyl acetate), 473.2% in *Curcuma longa* (ethyl acetate), and 284.5 in *Morus nigra* (ethyl acetate) were observed [95]. Tyrosinase inhibitory activities of the acetone, methanol, and hot water extracts from the fruiting bodies of *Lentinus* *lepideus* was found to be ranged from 9.7–58.8, 11.2–56.2, and 6.9–51.5%, respectively [96]. 

Inhibitory activities of *Bituminaria bituminosa* extracts were analyzed by Sarikurkcu et al. [97] and observed that the extracts exhibited no activity on tyrosinase, but the extracts showed various degrees of inhibitory activities on α-amylase and α-glucosidase. Inhibitory activities of the water extract (1233 μmol ACEs/g dry plant) on α-glucosidase were found to be greater than those of their inhibitory activities on α-amylase (53 μmol ACEs/g dry plant). 

## 4. Materials and Methods 

### 4.1. Plant Materials, Ascophyllum nodosum Extract (ANE), and Treatments Combinations 

Mung bean (*V. radiata* (L.) Wilczek) genotype viz., MH 215, was procured from the Pulses Section, Department of Genetics and Plant Breeding, CCS Haryana Agriculture University, Hisar (India). The *Ascophyllum nodosum* (L.) Le Jolis, (Seaweed) (Trade name: Biovita) extract (ANE) was purchased from PI industries, Rajasthan, India. Five concentrations ((A0) 0.00%, (A1) 0.01%, (A2) 0.05%, (A3) 0.10%, (A4) 0.50% *v/v*)), including control of ANE, were prepared by diluting the *A. nodosum* with sterilized, distilled water. Different concentrations of NaCl viz. (T0) 0 mM, (T1) 25 mM, (T2) 50 mM, (T3) 75 mM, (T4) 100 mM (*w/v*) were prepared in distilled water. The salt (NaCl) and ANE were used individually as well in combination for further studies in three replications/treatments (Table 9). 

### 4.2. Seed Sterilization and Germination of Seeds to Sprouts 

Seeds of identical size and color were surface sterilized with 0.1% HgCl_2_ for 4–5 min. Then, the seeds were washed thoroughly (2–3 times) with sterilized distilled water and then soaked in 20 mL of ANE (0.00%, 0.01%, 0.05%, 0.10%, and 0.50%), NaCl (25 mM, 50 mM, 75 mM, and100 mM) alone and with their combinations (Table 9) for 12 h in 100 mL beakers separately. A 12-hour soaking period was considered as 0 h of sprouting. After that, soaked seeds were kept in different Petri plates with ANE, NaCl, and their combinations (Table 9) for sprouting up to 36 h. Sprouts from 0 h, 24 h, and 36 h were taken for studying biochemical activities.

### 4.3. Preparation of Plant Extracts to Study the Various Biochemical Activities 

One gram of sprouts from different stages (0, 24, 36 h) and from different treatments was crushed in 2.0 mL of 80% methanol. The crushed sprouts were then stirred in orbital shaker at 200 rpm for 30 min at room temperature. The crushed material was then placed in water bath for 10 min at 60 °C and after that it was filtered two to three times with What-man filter paper 1. The filtrates were centrifuged at 10,000 rpm for 10 min. The supernatants were collected in tubes and the pellets were again washed with 2.0 mL of 80% methanol. The supernatant was pooled and used for estimation of antioxidants activities like:

- *DPPH free-radical-scavenging activity;*
- *ABTS free-radical-cation-scavenging activity;*
- *Total Phenol Content (TPC);*
- *Total Flavanoid Content (TFC);*
- *Reducing power assay;*
- *Alpha-amylase inhibition assay;*
- *Alpha-glucosidase inhibition assay;*
- *Tyrosinase inhibition activity.*

### 4.4. Estimation of DPPH (1,1 Diphenyl 2-Picryl Hydrazyl) Radical-Scavenging Activity 

The protocol of Sanchez-Moreno et al. [98] was followed to study the antioxidant activity in sprouts at different intervals. One mL DPPH solution (DPPH 6 × 10^−5^ M) was mixed with 250 μL of sprouts extracts of each treatment. The reaction mixture was shaken and kept for minimum 30 min in the dark at room temperature. The decrease in absorbance was measured at 517 nm. Ascorbic acid was used as a positive control for checking the free-radical-scavenging activities of the samples, where 250 μL of 80% methanol and 1.0 mL DPPH were mixed, kept for 30 min, and absorbance at 517 nm was recorded. The free-radical-scavenging activity in control and treatments samples at three time intervals (0 h, 24 h, and 36 h) was calculated as:

DPPH radical-scavenging activity (%) = [(A control) – (A Sample)]/A control × 100

where A control is the absorbance of control and A sample is the absorbance of sample.

### 4.5. Estimation of ABTS (2,2-azinobis (3-ethylbenzothiazoline-6-sulfonic acid) Scavenging Activity 

Re et al. [99] protocol was followed for estimation of ABTS scavenging activity, in which ABTS cation solution (7 mM) was prepared with potassium persulfate (2.45 mM) solution. The mixed solution was kept for 16 h in the dark at room temperature for complete oxidation. After that, ABTS cation was diluted with 95% ethanol (approximately 10 mL) to obtain 0.75 ± 0.05 absorbance at 734 nm. For estimation of treatment samples, 1.0 mL sprout extracts was mixed with 1.0 mL ABTS cation and for control, 1.0 mL of 80% methanol mixed with 1.0 mL of ABTS cation, and after 1 min, the absorbance was recorded at 734 nm. The free-radical-scavenging activity in control and treatments samples at three time intervals (0 h, 24 h, and 36 h) was calculated as:

ABTS scavenging activity (%) = [(A control) – (A Sample)]/A control × 100

where A control is the absorbance of control and A sample is the absorbance of sample.

### 4.6. Estimation of Total Phenolic Content (TPC) 

**Figure 5:**
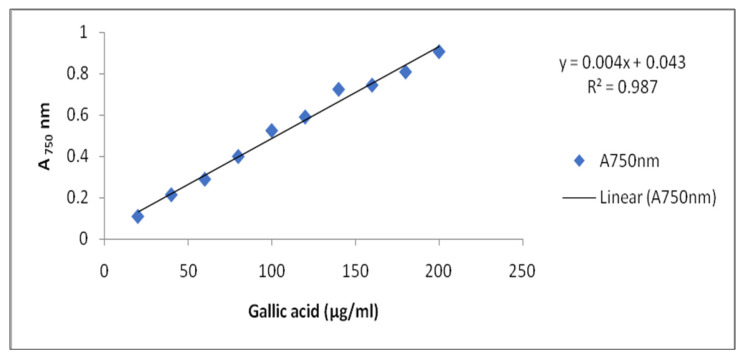
Standard curve of Gallic acid.

According to Kamtekar et al. [100], estimation of total phenolic content was conducted by Folin Ciocalteu’s method. One milliliter of each sprout’s extract was taken and then mixed with 5.0 mL distilled water plus 0.5 mL of Folin Ciocalteu’s reagent (2N). After 5 min, 2.5 mL of 20% sodium carbonate was added and allowed to incubate for 2 h at room temperature. Intense blue color was developed, which was measured at absorbance at 750 nm. The blank absorbance was set to 0.0 by taking 3.0 mL of 80% methanol. Gallic acid was used as standard for quantification of Total Phenolic Content (TPC) in controls and treatment samples. The calibration curve was plotted using standard gallic acid (20–200 μg/mL) and measured at 750 nm (Figure 5).

### 4.7. Estimation of Total Flavonoid Content (TFC) 

**Figure 6:**
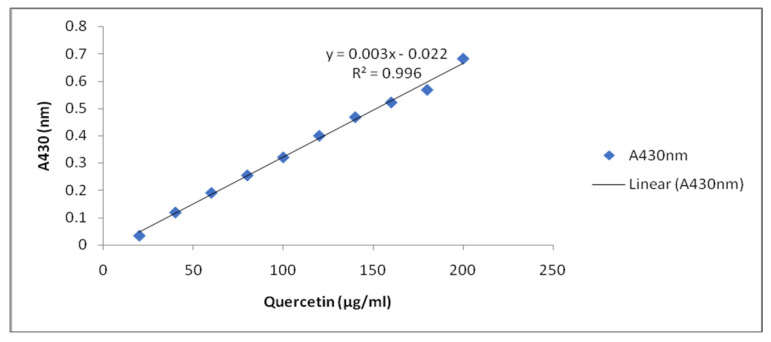
Standard curve of Quercetin.

Method of Djeridane et al. [101] was followed for estimation of TFC. The mixture was prepared by adding 1.0 mL of sprout extracts including control in 1.0 mL of aluminum chloride (AlCl_3_, 2%). The mixture was shaken vigorously and allowed to stand at room temperature for 30 min. The absorbance was recorded at 430 nm. TFC in extracts was estimated in mg Quercetin Equivalents (QE) per gram fresh weight, from the Quercetin standard calibration curve (Figure 6).

### 4.8. Estimation of Reducing Power Activity Assay 

The reducing powers of the samples were determined by protocol of Atmani et al. [102] with slight modifications. Sprout extracts (2.0 mL) were mixed with 2 mL of phosphate-buffered saline (0.2 M, pH 6.6) and 2 mL of potassium ferrocyanate (1%). The incubation for this mixture was set to 50 °C for 20 min. Then, 2.0 mL of trichloroacetic acid (10%) was added to this mixture. In a test tube, 2.0 mL from each of the above mixture was mixed with 2.0 mL of distilled water and 0.4 mL of 0.1% (*w/v*) ferric chloride. The absorbance was measured at 700 nm after 10 min. The increased absorbance of the reaction mixture indicated that the reducing power was high.

### 4.9. Estimation of α-Amylase (EC 3.2.1.1) Inhibition Assay 

The alpha-amylase inhibitory activity of the extract from sprouts was carried out according to Nickavar and Yousefian [103]. The starch (0.5%) solution was used as substrate and prepared by boiling starch in distilled water for 15 min. The DNS solution (20 mL 96 mM 3, 5-dinitrosalicylic acid, 12 g sodium potassium tartrate) in 8 mL of 2 M NaOH and 12 mL deionized water was used as the coloring reagent of reaction. One milliliter of extract and 1 ml of α-amylase enzyme was taken in test tubes and incubated at 25 °C for 30 min. Then, after taking out 1 mL from this mixture, 1 mL of the starch solution was added and the mixture was incubated at 25 °C for 3 min. Finally, 1 mL of the DNS solution was added. After that, tubes were covered and heated in water bath at 85 °C for 15 min. After cooling the tube, the reaction mixture was diluted with 9 mL distilled water and absorbance was recorded at 540 nm. For control, all procedure was same, except that sprout extract was replaced by 1 mL of DMSO. The absorbance at 540 nm was recorded at 0.472 for control sample. The percentage of alpha-amylase inhibition was calculated by using the formula:

α-Amylase inhibition activity (%) = [(A control) – (A Sample)]/A control × 100

where A control is the absorbance of control and A sample is the absorbance of sample.

### 4.10. Estimation of α-Glucosidase (EC 3.2.1.20) Inhibition Activity 

Protocol of Shai et al. [104] was used for α-glucosidase inhibition activity assay using 4-nitrophenyl α-d-glucopyranoside (PNPG) as substrate. Sprouts extracts (100 μL, 250 mg/mL) of all treatments were mixed with 50 μL (1 unit/mL) of α-glucosidase in sodium phosphate buffer (0.1 M, pH 6.8) and incubated for 20 min at 37 °C. After that, 100 μL of *p*-nitrophenyl-α-d-glucopyranoside (5.0 mM) was added. Then, 150 μL of sodium carbonate (0.2 M) was mixed and absorbance was taken at 405 nm. For control, buffer solution was taken in place of extract. The inhibition percentage was calculated using following formula:

α-Glucosidase inhibition activity (%) = [(A control) – (A Sample)]/A control × 100

where A control is the absorbance of control and A sample is the absorbance of sample.

### 4.11. Estimation of Tyrosinase (EC 1.14.18.1) Inhibition 

The protocol of Yao et al. [5] was followed for studying the tyrosinase inhibition activity. Sterile clean tubes were taken for each sample. A unit of 80 μL extract, 160 μL sodium phosphate buffer (50 mM, pH 6.6), (31 unit/mL) of tyrosinase enzyme, and 80 μL of L-dopa (2.5 mM) were added for all treated concentrations including control. The reaction solutions were incubated for 30 min at room temperature and the absorbance was measured at 475 nm. The control group had all the constituents except for the plant extract. The percentage inhibitory activity was calculated by using the formula:

Tyrosinase inhibition activity (%) = [(A control) – (A Sample)]/A control × 100

where A control is the absorbance of control and A sample is the absorbance of sample.

### 4.12. Statistical Analysis 

The obtained data were subjected to the statistical analysis of variance procedure using two-way-ANOVA of the Microsoft Excel. Values are expressed as an average of three measurements ± standard deviation (SD). A two-way analysis of variance was employed to estimate the significance of the observed difference due to ANE application to salinity-stressed plants and the changes induced due to salinity stress and ANE applications at *p* ≤ 0.05 level of significance using Tukey’s Test.

## 5. Conclusions

The results of this study are important because a significant increase in antioxidants during the germination of *V. radiata* can make them more desirable for consumption. In this fast-paced and convenient society, a sprout could be a quick and inexpensive way to get invaluable nutrition in the diet. Therefore, such an ability could be claimed to prevent many human-health-related diseases, such as diabetes, cancer, aging, atherosclerosis, etc. that are associated with free radicals. Salinity negatively regulates the anti-tyrosinase activity of sprouts of *V. radiata* seeds. The anti-tyrosinase, α-glucosidase, α-amylase activity, and polyphenols were found to marginally increase with time from 0 to 36 h. An increase in salinity to the concentration of 100 mM NaCl completely abolished α-amylase activity after 0, 24, and 36 h of treatment. The ANE treatment was found to reverse the salinity-mediated decrease of the flavonoid content. The increasing trend was found for phenol accumulation when ANE was used in the saline environment. *A. nodosum* extract is natural, environmentally friendly, safe, and can be used as one of the strategies to decline plant’s stress at low levels and enhance the antioxidant activities in *V. radiata* sprouts, thus increasing its potential to be developed as an antioxidant-based functional food.

## Figures and Tables

**Table 1:** Two-way analysis results of DPPH assay of sprouts of *V. radiata* under salt stress in the presence of *A. nodosum* extract.

**ANOVA: Two-Factor 0 h for DPPH Radical-Scavenging Activity**						
	***SS***	***df***	***MS***	***F***	***p-Value***	***F crit***
Salinity	235.7898	4	58.94746	9.115549	0.000491	3.006917
ANE	735.0457	4	183.7614	28.4166	4.31 × 10^−7^	3.006917
Error	103.4671	16	6.466693			
Total	1074.303	24				
**ANOVA: Two-Factor 24 h for DPPH Radical-Scavenging Activity**						
	***SS***	***df***	***MS***	***F***	***p-Value***	***F crit***
Salinity	548.9184	4	137.2296	8.194715	0.000854	3.006917
ANE	1144.414	4	286.1036	17.08478	1.26 × 10^−5^	3.006917
Error	267.9378	16	16.74611			
Total	1961.27	24				
**ANOVA: Two-Factor 36 h for DPPH Radical-Scavenging Activity**						
	***SS***	***df***	***MS***	***F***	***p-Value***	***F crit***
Salinity	369.0484	4	92.2621	4.21749	0.016093	3.006917
ANE	808.8904	4	202.2226	9.24401	0.000456	3.006917
Error	350.0171	16	21.87607			
Total	1527.956	24				

**Table 2:** Two-way analysis results of ABTS assay of sprouts of *V. radiata* under salt stress in the presence of *A. nodosum* extract.

**ANOVA: Two-Factor (ABTS Activity Assay at 0 h)**						
	***SS***	***df***	***MS***	***F***	***p-Value***	***F crit***
Salinity	5040.449617	4	1260.112	33.12243	1.48 × 10^−7^	3.006917
ANE	1752.822066	4	438.2055	11.51836	0.000135	3.006917
Error	608.7053571	16	38.04408			
Total	7401.977041	24				
**ANOVA: Two-Factor (ABTS Activity Assay at 24 h)**						
	***SS***	***df***	***MS***	***F***	***p-Value***	***F crit***
Salinity	7000.749362	4	1750.187	53.64041	4.54 × 10^−9^	3.006917
ANE	1530.484694	4	382.6212	11.72672	0.000122	3.006917
Error	522.0503827	16	32.62815			
Total	9053.284439	24				
**ANOVA: Two-Factor (ABTS Activity Assay at 36 h)**						
	***SS***	***df***	***MS***	***F***	***p-Value***	***F crit***
Salinity	1723.740434	4	430.9351	6.029559	0.003718	3.006917
ANE	6299.394133	4	1574.849	22.03497	2.41 × 10^−6^	3.006917
Error	1143.526786	16	71.47042			
Total	9166.661352	24				

**Table 3:** Two-way analysis results for phenolic content of sprouts of *V. radiata* under salt stress in the presence of *A. nodosum* extract.

**ANOVA: Two-Factor 0 h for Phenol Content**						
	***SS***	***df***	***MS***	***F***	***p-Value***	***F crit***
Salinity	31383.27	4	7845.818	9.992317	0.000299	3.006917
ANE	39683.6	4	9920.901	12.63511	7.91 × 10^−5^	3.006917
Error	12562.96	16	785.1851			
Total	83629.84	24				
**ANOVA: Two-Factor 24 h for Phenol Content**						
	***SS***	***df***	***MS***	***F***	***p-Value***	***F crit***
Salinity	57971.77	4	14492.94	11.59064	0.00013	3.006917
ANE	44068.22	4	11017.05	8.810816	0.00059	3.006917
Error	20006.42	16	1250.401			
Total	122046.4	24				
**ANOVA: Two-Factor 36 h for Phenol Content**						
	***SS***	***df***	***MS***	***F***	***p-Value***	***F crit***
Salinity	74439.07	4	18609.77	19.13987	6.07 × 10^−6^	3.006917
ANE	120094.4	4	30023.59	30.87881	2.42 × 10^−7^	3.006917
Error	15556.86	16	972.3038			
Total	210090.3	24				

**Table 4:** Two-way analysis results for the flavonoid content of sprouts of *V. radiata* under salt stress in the presence of *A. nodosum* extract.

**ANOVA: Two-Factor 0 h for Total Flavonoid Content**						
	***SS***	***df***	***MS***	***F***	***p-Value***	***F crit***
Salinity	8108.798	4	2027.199	17.61242	1.04 × 10^−5^	3.006917
ANE	1498.642	4	374.6605	3.255071	0.039183	3.006917
Error	1841.609	16	115.1006			
Total	11449.05	24				
**ANOVA: Two-Factor 24 h for Total Flavonoid Content**						
	***SS***	***df***	***MS***	***F***	***p-Value***	***F crit***
Salinity	2140.035	4	535.0087	4.115023	0.017621	3.006917
ANE	5349.083	4	1337.271	10.28563	0.000255	3.006917
Error	2080.216	16	130.0135			
Total	9569.334	24				
**ANOVA: Two-Factor 36 h for Total Flavonoid content**						
	***SS***	***df***	***MS***	***F***	***p-Value***	***F crit***
Salinity	5694.78	4	1423.695	9.427506	0.00041	3.006917
ANE	15312.17	4	3828.042	25.34875	9.42 × 10^−7^	3.006917
Error	2416.24	16	151.015			
Total	23423.19	24				

**Table 5:** Two-way analysis results for reducing power of sprouts of *V. radiata* under salt stress in the presence of *A. nodosum* extract.

**ANOVA: Two-Factor 0 h for Reducing Power**						
	***SS***	***df***	***MS***	***F***	***p-Value***	***F crit***
Salinity	0.023728	4	0.005932	1.631845	0.214859	3.006917
ANE	0.077387	4	0.019347	5.322112	0.006398	3.006917
Error	0.058163	16	0.003635			
Total	0.159279	24				
**ANOVA: Two-Factor 24 h for Reducing Power**						
	***SS***	***df***	***MS***	***F***	***p-Value***	***F crit***
Salinity	0.204465	4	0.051116	7.332341	0.001488	3.006917
ANE	0.328389	4	0.082097	11.77638	0.000119	3.006917
Error	0.111541	16	0.006971			
Total	0.644395	24				
**ANOVA: Two-Factor 36 h for Reducing Power**						
	***SS***	***df***	***MS***	***F***	***p-Value***	***F crit***
Salinity	0.341818	4	0.085455	34.10045	1.2 × 10^−7^	3.006917
ANE	0.296523	4	0.074131	29.58171	3.26 × 10^−7^	3.006917
Error	0.040095	16	0.002506			
Total	0.678437	24				

**Table 6:** Two-way analysis results for α-amylase activity of sprouts of *V. radiata* under salt stress in the presence of *A. nodosum* extract.

**ANOVA: Two-Factor 0 h for α-Amylase**						
	***SS***	***df***	***MS***	***F***	***p-Value***	***F crit***
Salinity	0.10878	4	0.027195	34.98703	1 × 10^−7^	3.006917
ANE	0.122265	4	0.030566	39.32411	4.36 × 10^−8^	3.006917
Error	0.012437	16	0.000777			
Total	0.243482	24				
**ANOVA: Two-Factor 24 h for α-Amylase**						
	***SS***	***Df***	***MS***	***F***	***p-Value***	***F crit***
Salinity	0.273032	4	0.068258	26.80128	6.44 × 10^−7^	3.006917
ANE	0.233946	4	0.058487	22.96455	1.83 × 10^−6^	3.006917
Error	0.040749	16	0.002547			
Total	0.547728	24				
**ANOVA: Two-Factor 36 h for α-Amylase**						
	***SS***	***Df***	***MS***	***F***	***p-Value***	***F crit***
Salinity	0.204599	4	0.05115	15.27371	2.53 × 10^−5^	3.006917
ANE	0.229352	4	0.057338	17.12158	1.24 × 10^−5^	3.006917
Error	0.053582	16	0.003349			
Total	0.487533	24				

**Table 7:** Two-way analysis results for α-glucosidase activity of sprouts of V. radiata under salt stress in the presence of *A. nodosum* extract.

**ANOVA: Two-Factor 0 h for α-Glucosidase**						
	***SS***	***df***	***MS***	***F***	***p-Value***	***F crit***
Salinity	2983.619	4	745.9048	75.32564	3.59 × 10^−10^	3.006917
ANE	2782.22	4	695.555	70.24103	6.08 × 10^−10^	3.006917
Error	158.4384	16	9.902402			
Total	5924.278	24				
**ANOVA: Two-Factor 24 h for α-Glucosidase**						
	***SS***	***df***	***MS***	***F***	***p-Value***	***F crit***
Salinity	4561.108	4	1140.277	115.4178	1.38 × 10^−11^	3.006917
ANE	1847.639	4	461.9098	46.75409	1.25 × 10^−8^	3.006917
Error	158.073	16	9.87956			
Total	6566.82	24				
**ANOVA: Two-Factor 36 h for α-Glucosidase**						
	***SS***	***df***	***MS***	***F***	***p-Value***	***F crit***
Salinity	3599.931	4	899.9827	229.8666	6.49 × 10^−14^	3.006917
ANE	1206.688	4	301.6719	77.05071	3.03 × 10^−10^	3.006917
Error	62.64382	16	3.915239			
Total	4869.263	24				

**Table 8:** Two-way analysis results for anti-tyrosinase activity of sprouts of *V. radiata* under salt stress in the presence of *A. nodosum* extract.

**ANOVA: Two-Factor 0 h for Anti-Tyrosinase Activity**						
	***SS***	***df***	***MS***	***F***	***p-Value***	***F crit***
Salinity	94.05033	4	23.51258	9.066553	0.000505	3.006917
ANE	136.6049	4	34.15122	13.16886	6.19 × 10^−5^	3.006917
Error	41.49331	16	2.593332			
Total	272.1485	24				
**ANOVA: Two-Factor 24 h for Anti-Tyrosinase Activity**						
	***SS***	***df***	***MS***	***F***	***p-Value***	***F crit***
Salinity	230.9833	4	57.74583	10.85751	0.00019	3.006917
ANE	22.01747	4	5.504367	1.034944	0.419617	3.006917
Error	85.09627	16	5.318517			
Total	338.0971	24				
**ANOVA: Two-Factor 36 h for Anti-Tyrosinase Activity**						
	***SS***	***df***	***MS***	***F***	***p-Value***	***F crit***
Salinity	85.82178	4	21.45545	19.5978	5.21 × 10^−6^	3.006917
ANE	164.1451	4	41.03628	37.4833	6.15 × 10^−8^	3.006917
Error	17.51661	16	1.094788			
Total	267.4835	24				

**Table 9:** Experimental designs of different treatments combinations.

	NaCl (mM) 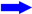	0 (T0)	25 (T1)	50 (T2)	75 (T3)	100 (T4)
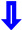 ANE (%)						
0.00 (A0)		0 + 0.00 (T0A0)	25 + 0.00 (T1A0)	50 + 0.00 (T2A0)	75 + 0.00 (T3A0)	100 + 0.00 (T4A0)
0.01 (A1)		0 + 0.01 (T0A1)	25 + 0.01 (T1A1)	50 + 0.01 (T2A1)	75 + 0.01 (T3A1)	100 + 0.01 (T4A1)
0.05 (A2)		0 + 0.05 (T0A2)	25 + 0.05 (T1A2)	50 + 0.05 (T2A2)	75 + 0.05 (T3A2)	100 + 0.05 (T4A2)
0.10 (A3)		0 + 0.10 (T0A3)	25 + 0.10 (T1A3)	50 + 0.10 (T2A3)	75 + 0.10 (T3A3)	100 + 0.10 (T4A3)
0.50 (A4)		0 + 0.50 (T0A4)	25 + 0.50 (T1A4)	50 + 0.50 (T2A4)	75 + 0.50 (T3A4)	100 + 0.50 (T4A4)

## Data Availability

All data, tables, figures and results in paper are our own and original.
